# Development of a species-specific TaqMan-MGB real-time PCR assay to quantify *Olsenella scatoligenes* in pigs offered a chicory root-based diet

**DOI:** 10.1186/s13568-018-0627-y

**Published:** 2018-06-16

**Authors:** Xiaoqiong Li, Bent Borg Jensen, Ole Højberg, Samantha Joan Noel, Nuria Canibe

**Affiliations:** 1 Department of Animal Science, Faculty of Science and Technology, Aarhus University, Blichers Allé 20, P. O. Box 50, 8830 Tjele, Denmark; 2 Present Address: Institute of Food Science, Zhejiang Academy of Agricultural Sciences, Hangzhou, 310021 China

**Keywords:** Chicory root, MGB probe, *Olsenella scatoligenes*, qPCR, Skatole, TaqMan

## Abstract

*Olsenella scatoligenes* is the only skatole-producing bacterium isolated from the pig gut. Skatole, produced from microbial degradation of *l*-tryptophan, is the main contributor to boar taint, an off-odor and off-flavor taint, released upon heating meat from some entire male pigs. An appropriate method for quantifying *O. scatoligenes* would help investigating the relationship between *O. scatoligenes* abundance and skatole concentration in the pig gut. Thus, the present study aimed at developing a TaqMan-MGB probe-based, species-specific qPCR assay for rapid quantification of *O. scatoligenes*. The use of a MGB probe allowed discriminating *O. scatoligenes* from other closely related species. Moreover, the assay allowed quantifying down to three target gene copies per PCR reaction using genomic DNA-constructed standards, or 1.5 × 10^3^ cells/g digesta, using *O. scatoligenes*-spiked digesta samples as reference standards. The developed assay was applied to assess the impact of dietary chicory roots on *O. scatoligenes* in the hindgut of pigs. *Olsenella scatoligenes* made up < 0.01% of the microbial population in the pig hindgut. Interestingly, the highest number of *O. scatoligenes* was found in young entire male pigs fed high levels of chicory roots. This indicates that the known effect of chicory roots for reducing skatole production is not by inhibiting the growth of this skatole-producing bacterium in the pig hindgut. Accordingly, the abundance of *O. scatoligenes* in the hindgut does not seem to be an appropriate indicator of boar taint. The present study is the first to describe a TaqMan-MGB probe qPCR assay for detection and quantification of *O. scatoligenes* in pigs.

**Electronic supplementary material:**

The online version of this article (10.1186/s13568-018-0627-y) contains supplementary material, which is available to authorized users.

## Introduction

Skatole (3-methylindole) is the main compound responsible for boar taint, which is an off-odor and off-flavor meat trait released upon heating of meat from some entire male pigs (Wesoly and Weiler 2012). Boar tainted meat is disagreeable to most consumers and has therefore a negative economic impact for the meat industry (Jensen et al. 2014). Skatole is produced by anaerobic microbial degradation of l-tryptophan (TRP) via decarboxylation of indol-3-acetic acid (IAA) in the hindgut of pigs. It is produced in both sexes, but increased in some entire male pigs, probably due to a reduced skatole metabolism in the liver of these animals (Babol et al. 1999; Zamaratskaia et al. 2004). Dietary supplementation with easily fermentable carbohydrates with low ileal digestibility has been shown to reduce skatole production in the hindgut (Jensen et al. 1995; Knarreborg et al. 2002; Rideout et al. 2004; Lösel and Claus 2005; Hansen et al. 2006; Øverland et al. 2011; Vhile et al. 2012; Zammerini et al. 2012). The most effective carbohydrate seems to be purified inulin or inulin-rich feed components like chicory root or Jerusalem artichoke (Jensen et al. 2014).

Whereas many bacteria are able to deaminate TRP to IAA (Patten et al. 2013), only few bacteria have been reported to catalyze the step from IAA to skatole. *Clostridium scatologenes* and *Clostridium drakei* SL1, isolated from soil and acidic sediment, respectively, are able to produce skatole from TRP via IAA (Whitehead et al. 2008); while *Olsenella uli* and *Olsenella scatoligenes* have been shown to produce skatole from IAA, but are unable to convert TRP to skatole (Li et al. 2015). It has been estimated that skatole-producing bacteria represent less than 0.01% of the total intestinal microbiota of the pig (Jensen and Jensen 1998). Of the skatole-producing bacteria, only *O. scatoligenes* has so far been isolated from the pig gastrointestinal (GI)-tract (Li et al. 2015). Other studies have revealed that dietary fiber enhances *Olsenella* abundance in pigs (Haenen et al. 2013) and in mice (Mao et al. 2015). However, whether the impact of chicory root on skatole production is related to a change in the population of *O. scatoligenes* in the GI-tract of pigs has not been elucidated.

Not only detection and identification of skatole-producing bacteria, but also establishment of their density related to skatole production in the hindgut and changes due to dietary fermentable carbohydrates are important in order to design strategies aiming at reducing boar taint. This paper describes a TaqMan minor groove binder (MGB) qPCR assay targeting the 16S rRNA gene of *O. scatoligenes* SK9K4^T^ to quantify the *O. scatoligenes* species. The specificity, sensitivity, and stability of this method were validated from standard DNA curves. The established TaqMan-MGB qPCR assay was applied to detect and compare *O. scatoligenes* densities in the hindgut of pigs fed diets with or without chicory roots.

## Materials and methods

### Ethics statement

All animal experimental procedures were carried out in accordance with the Danish Ministry of Justice, Law No. 253 of March 2013 concerning experiments with animals and care of experimental animals. The study was approved by the Danish Animal Experimentation Board (License No. 2010/561-1914).

### Experimental protocol and sampling

From an initial total of 36 pigs used in another study, 24 [Duroc × (Danish Landrace × Yorkshire)] pigs with an initial body weight of ~ 25 kg and aged 7 weeks (13 entire males and 11 females) were included in the current study. Pigs were fed either a standard Danish grower diet based on wheat, barley, and soybean meal (control); or the same diet in which part of the wheat was substituted with 25% chicory root (chicory). The ingredient composition and chemical analyses of the diets are shown in Additional file 1: Table S1. Pigs (n = 18 in each treatment) were housed in six pens (three pens with six animals per treatment) with partially slatted concrete floor and a few handfuls of woodchips in the resting area. Pigs were allowed ad libitum access to the feed and water. After 5 weeks on the experimental diets and an average bodyweight of ~ 60 kg, two pigs from each pen were sacrificed with a captive bolt pistol followed by exsanguination. Immediately after, the large intestine was removed and divided into five sections: cecum, and four equally long segments of the colon including the rectum. Approximately 5 g digesta from the cecum and the third segment of the colon were collected, and aliquots immediately frozen and stored at − 20 °C for analyses. After 4 more weeks on the diet, another two pigs from each pen were killed. Samples from these pigs are not included in the present study. The remaining pigs (2 per pen, 6 per treatment) continued on the same feed for another 4 weeks, reaching a body weight of ~ 120 kg, and were killed and sampled following the same procedure as described for those killed at ~ 60 kg body weight. The cecum and colon samples from the 24 pigs (12 per treatment) were included in the present study.

### Extraction of DNA from digesta

Cecal digesta samples were freeze dried at − 55 °C for 72 h (Scanvac Coolsafe, model no. 55-4, Labogene, Lillerød, Denmark) prior to DNA extraction. Microbial DNA from approximately 200 mg wet colonic digesta and 50 mg freeze-dried cecal digesta (equal to around 500 mg wet weight) was extracted using the QIAamp^®^ Fast DNA Stool mini Kit (Qiagen, Hilden, Germany) according to the manufacturer’s instructions for pathogen detection. Briefly, digesta samples were homogenized in the buffer InhibitEX and heated at 95 °C for 5 min to lyse bacterial cells. The lysates were treated with proteinase K and buffer AL at 70 °C for 10 min to remove protein and polysaccharides. DNA was precipitated by ethanol, applied to a column provided in the kit followed by washes with buffers AW1 and AW2, and then dissolved in buffer AE and stored at − 20 °C.

### Bacteria strains, growth conditions, and genomic DNA extraction

The type strain of *O. scatoligenes*, SK9K4 DSM 28304^T^ served as a positive control. The type strains of four closely related species: *O. uli* DSM 7084^T^, *O. profusa* DSM 13989^T^, *O. umbonata* DSM 22620^T^, and *Atopobium parvulum* DSM 20469^T^, which were obtained from German collection of microorganisms and cell cultures (DSMZ), were used as negative controls. Moreover, strains dominating in the pig gut collected in our laboratory: *Bacteroides* sp. DJF_B097, *Lactobacillus* sp. DJF_B156, *Ruminococcus* sp. DJF_VR87, *Enterococcus faecalis* DJF_O03, *Megasphaera elsdenii* DJF_RP06, *Prevotellaceae* sp. DJF_LS10, and *Roseburia* sp. DJF_RR73 were also used as negative controls. All strains were cultured anaerobically in modified Peptone-Yeast extract with Glucose medium for 48 h at 37 °C as described previously (Li et al. 2015). Genomic DNA (gDNA) from the different reference strains was extracted using a Maxwell 16S DNA purification kit (Promega, Madison, Wisconsin, USA) and automated DNA purification was performed on a Maxwell 16 Instrument (Promega, Madison WI, USA) according to the technical manual provided by the manufacturer.

### DNA yield and quality

Quantity and quality of DNA extracted from digesta samples and pure culture samples was measured by reading the whole absorption spectrum (220–750 nm) with a NanoDrop 1000 spectrophotometer (Thermo Fisher Scientific, Waltham, MA, USA), and the DNA quality assessed using absorbance ratio at both 260/280 and 230/260 nm. The DNA concentration in each sample was then quantified with the Qubit fluorometer 3.0 (Life Technologies, Grand Island, NY, USA). The instrument was calibrated with the Qubit^®^ dsDNA HS Assay kit (accurate for initial sample concentration between 10 pg/µL and 100 ng/µL) according to the manufacturer’s instructions (Simbolo et al. 2013).

### Species-specific primer and probe design

**Fig. 1:**
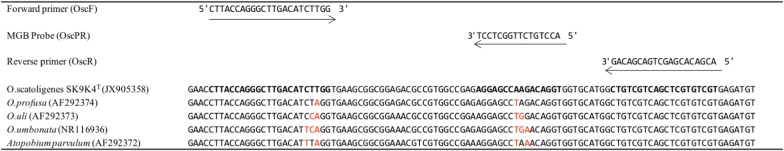
Sequence targeted by the *O. scatoligenes*-specific MGB probe and primers, taking into account analogous sequences in the closely related *Olsenella* species and *Atopobium* species. Mismatches are marked in red

AlleleID^®^ 6.0 (Premier Biosoft, Palo Alto, CA, USA) and the Primer Express Software 3.0 (Life Technologies) were combined to design a primer–probe pair specific for *O. scatoligenes* SK9K4^T^ targeting the hypervariable region V6 of the 16S ribosomal RNA gene sequence (Chakravorty et al. 2007). Figure 1 shows the sequence targeted by the *O. scatoligenes*-specific TaqMan MGB probe and primers, and part of homologous 16S rRNA gene sequences from closely related *Olsenella* species and *A. parvulum* strains. The basic local alignment search tool (BLASTn) (http://www.ncbi.nlm.nih.gov/BLAST) and the ribosomal database project (RDP) (https://rdp.cme.msu.edu/probematch/search.jsp) were used for preliminary assessments of oligonucleotide specificity prior to synthesis. The designed primers OscF: 5′-CTTACCAGGGCTTGACATCTTGG-3′ (positions: 949–971) and OscR: 5′-ACGACACGAGCTGACGACAG -3′ (positions: 1024–1043) were obtained from DNA Technology A/S (Aarhus, Denmark). The TaqMan probe was labeled with 6-carboxyfluorescein (FAM) at the 5′ end and a nonfluorescent quencher (NFQ) with MGB ligands was used as the quencher at the 3′ end. OscPR: 5′-6-FAM-ACCTGTCTTGGCTCCT-MGB-NFQ-3′ (positions: 999–1014) was purchased from Applied Biosystems (Life Technologies, Loughborough, UK).

### PCR procedure

To validate each new set of primers, a conventional PCR was performed followed by SYBR Green qPCR. The optimal primer pairs were then selected based on specificity and efficiency of amplification. Next, the TaqMan qPCR was carried out with the primer-MGB probe pair.

PCR was performed on a DOPPIO thermocycler (VWR International, USA) with a PCR program: 94 °C for 10 min, denaturation at 94 °C for 30 s, annealing at 62 °C for 30 s, and elongation at 72 °C for 30 s. Thirty cycles were conducted, followed by a final elongation step at 72 °C for 10 min. The mix consisted of 0.5 µL of the forward primer and the reverse primer, each at a concentration of 10 nmol/µL, 1 µL dNTP (5 nmol/µL), 0.5 µL DyNazymeTM II DNA polymerase (2 U/µL), 2.5 µL DyNazyme 10× buffer (Finnzymes, Espo, Finland), and 2 µL template DNA to a final volume of 25 µL per reaction. The specificity of the primer pairs was confirmed by attempting to amplify an extensive set of closely related species and pig dominant gut bacterial species as controls. The PCR products were subjected to electrophoresis on a 1.5% agarose gel. DNA was stained with GelRed™ Nucleic Acid Gel Stain (Biotium, Fremont, CA, USA) and viewed under long-wavelength UV light.

Quantitative real-time PCR was performed using a ViiA™ 7 Real-time PCR System (Applied Biosystems, Foster City, CA, USA) associated with the ViiA™ 7 RUO software version 1.2.1 (Life Technologies, Taastrup, Denmark). For SYBR Green qPCR, all PCR experiments were carried out in duplicate with a reaction volume of 10 µL, consisting of 5 µL of 2× Maxima SYBR Green/ROX qPCR master mix (Thermo Fisher Scientific, Roskilde, Denmark), 300 nM of each primer, and 2 µL of DNA template. The qPCR program was as follows: 50 °C for 2 min, 95 °C for 10 min, followed by 40 cycles at 95 °C for 15 s, 62 °C for 30 s, and 72 °C for 30 s. The same set of reference strains was used as a negative control. A non-template control of nuclease-free water was included in each run. To determine the specificity of PCR reactions, melt curve analysis was carried out after amplification.

For the TaqMan qPCR, amplifications were carried out in triplicate in a total volume of 10 µL consisting of 5.0 µL TaqMan Universal master mix (Applied Biosystems, Foster City, CA, USA), 300 nM of each primer, 200 nM of TaqMan MGB probe, and 2 µL of gDNA template. For digesta samples, about 10 ng DNA measured by Qubit^®^ fluorometer was used. The qPCR program was: hold 2 min at 50 °C, followed by 10 min at 95 °C, then 40 cycles of 15 s at 95 °C, and 1 min at 60 °C. The same extensive set of reference strains was used as a negative control. Every qPCR run included a negative control and a standard curve consisting of SK9K4^T^ gDNA with known concentrations.

### Preparation of PCR standards for *Olsenella scatoligenes*

#### gDNA standard

A tenfold dilution series from 3 × 10^7^ to 3 × 10^0^ copies per reaction of strain SK9K4^T^ gDNA was set up to generate gDNA standards for absolute quantification of *O. scatoligenes*. The mass of SK9K4^T^ genome (*m)* was calculated with the following formula (*m *=(*n *×* M*)/*N*_*A*_) (http://www.appliedbiosystems.com/support/tutorials/pdf/quant_pcr.pdf), in which *n* is the genome size (bp), *N*_A_ is Avogadro’s number (6.023 × 10^23^ bp/mol), and *M* is the average molecular weight of a double-stranded DNA molecule (660 g/mol). The genome size of the SK9K4^T^ as determined by the high-throughput sequencing Illumina HiSeq 2000 platform [Beijing Genomics Institute (BGI), Shenzhen, China] was 2.47 Mbp. There is only one copy of the 16s rRNA gene located in the chromosome of SK9K4^T^ (Li et al. 2016a). Therefore, it was assumed that one DNA copy is equivalent to one bacterial cell. SK9K4^T^ gDNA extractions were measured by the Qubit^®^ fluorometer to determine the appropriate amount (40.65 ng/uL equivalent to 1.5 × 10^7^ copies/µL) needed for the initial dilution steps. To generate standard curves, the Ct values were plotted against the logarithm of the corresponding template copy numbers. Each standard curve was generated by linear regression of the plotted points. PCR amplification efficiency (*E*) was calculated from the slope of the standard curve: *E* = 10(^−1/slope^) − 1. The limit of quantification (LOQ) was obtained from gDNA standard curves.

#### Reference standard

Reference standard curves based on a serial dilution of DNA after extraction from 200 mg of cecal or colonic digesta spiked with 1.5 × 10^10^ SK9K4^T^ cells/g were constructed (ranging from 3 × 10^7^ to 3 × 10^0^ cells per reaction). The initial pure culture stock of SK9K4^T^ was prepared by concentrating a 48-h-old culture in physiological salt solution. The cell number of the stock (6.0 × 10^10^ cells/mL) was determined by microscopic cell counting with the Petroff-Hausser Counting Chamber. The LOQ in intestinal content was obtained from reference standard curves.

### MBG TaqMan real-time PCR reproducibility

The reproducibility of the assay was demonstrated by evaluating the variability of the Ct values obtained after amplification of tenfold dilutions of the SK9K4^T^ gDNA standards ranging from 3 × 10^7^ to 3 × 10^0^ copies per reaction in triplicate intra- and inter-run. The coefficient of variation (CV) was determined for each of the concentrations of DNA.

### DNA recovery assay

For digesta samples, the efficiency of DNA extraction was determined by DNA recovery rate and PCR inhibitor reduction during DNA extraction. The recovery rate was assessed by adding quantified reference strain *O. scatoligenes* SK9K4^T^ to colonic digesta samples. Specifically, 200 mg colonic digesta samples were spiked with 3 × 10^2^ to 3 × 10^9^ cells of SK9K4^T^, giving final spiked concentrations of 1.5 × 10^3^ to 1.5 × 10^10^ cells/g digesta. The DNA recovery rate of extraction from the digesta samples was assessed by first using the gDNA standard curve to convert the Ct to *O. scatoligenes* genomic copies/reaction, then back-calculating with volumes and dilutions to determine *O. scatoligenes* cells/reaction for the spiked gut digesta samples. Finally, recovery rate was calculated as the quotient of recovered copies and spiked cells as described by Wattanaphansak et al. (2010).

### Quantification of *Olsenella scatoligenes* and skatole concentration in gut digesta samples

To illustrate the quantification method, samples from a study on the effect of chicory roots on skatole production in the hindgut of entire male and female pigs at two different ages were used. To determine and compare *O. scatoligenes* numbers in different treatment groups, all real-time PCR data were calculated against reference standards, and log *O. scatoligenes* cells/g digesta (wet weight) were calculated and presented. The microbial DNA inputs were normalized according to the DNA concentration determined by Qubit^®^ fluorometer (Thermo Fisher Scientific, Roskilde, Denmark) to ensure that the *O. scatoligenes* cells in digesta were compared at the same DNA extraction level during the experiment. The concentration of phenolic- and indolic compounds in digesta was analyzed by high performance liquid chromatography (HPLC) as described by Knarreborg et al. (2002).

### Data analysis

The SAS statistical software package, version 9.3 (SAS Institute, Inc., Cary, NC), was used for statistical analyses. Data were analyzed using the software’s GLM procedure. The statistical model included the fixed effects of diet, sex, age, and their interactions. Differences between least square means were compared using Tukey–Kramer test (Littell et al. 2002). A *p* value of < 0.05 was considered statistically significant.

## Results

### DNA yield and purity

Both NanoDrop^®^ spectrophotomer and Qubit^®^ fluorometer were used to measure the amount of extracted DNA. The DNA quality and purity was assessed spectrophotometrically. The A260/A280 ratio was good in most of the extracts (1.8–2.0). DNA concentrations measured by NanoDrop^®^ were higher than those measured by Qubit^®^ (Table 1). Measured by NanoDrop^®^, cecal and colonic digesta DNA extraction using QIAamp^®^ Fast DNA Stool mini Kit yielded an average concentration of DNA of 20.9 ± 8.4 and 58.7 ± 13.2 µg/g wet weight digesta, respectively. The same DNA samples quantified with the Qubit^®^ system showed somehow lower DNA concentrations (i.e. average DNA concentrations of 1.0 ± 0.8 and 5.1 ± 2.1 µg/g from cecal and colonic digesta, respectively). According to Simbolo et al. (2013), Qubit proves highly reproducible, showing consistent results, and highly correlated to qPCR measurements. Thus, DNA concentrations read from Qubit were used to normalize DNA template concentration in the TaqMan real-time PCR studies.

**Table 1:** Comparison of microbial DNA concentration from pig digesta samples measured by NanoDrop^®^ spectrophotomer and Qubit^®^ fluorometer quantitation platforms

Sample	NanoDrop^®^ (µg/g)	Qubit^®^ (µg/g)
Cecal digesta (n = 24)	20.9 ± 8.4	1.0 ± 0.8
Colonic digesta (n = 47)	58.7 ± 13.2	5.1 ± 2.1

### Specificity of TaqMan-MGB qPCR for *Olsenella scatoligenes*

**Fig. 2:**
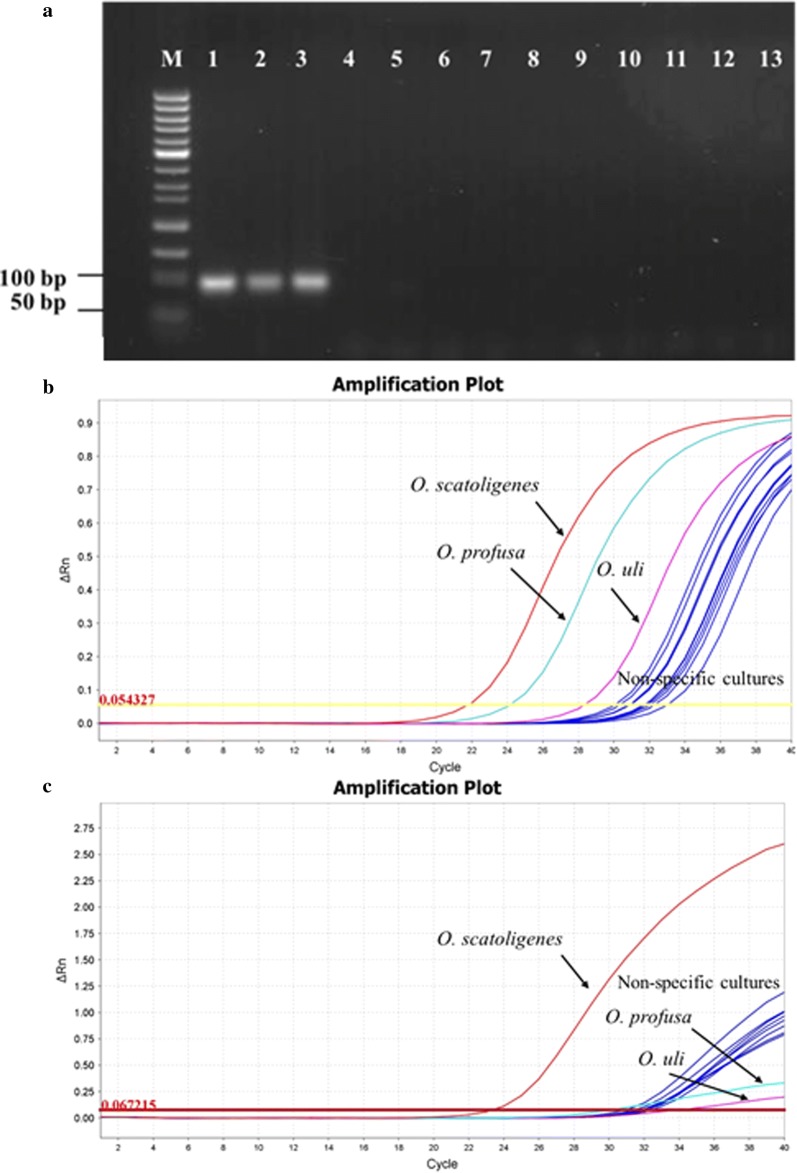
Specificity of the primer pair alone and TaqMan primer–probe pair for *Olsenella scatoligenes* SK9K4^T^ using genomic DNA targeting a specific region of the 16s rRNA gene sequence. **a** Conventional PCR with the primer pair alone. 1, *O. scatoligenes*; 2, *O. uli*; 3, *O. profusa*; 4–12, non-specific cultures; 13, distilled water. **b** SYBR Green real-time PCR with the primer pair alone. **c** TaqMan-MGB real-time PCR with the primer–probe pair

The specificity of the primer pairs and the TaqMan-MBG probe was first assessed by BLASTn and the RDP database. A probe match search in the RDP database showed that the sequence of MGB OscP-1014 targeting the V6 area of 16S rRNA matched exactly and only with *O. scatoligenes* species [including the strains *O.* sp. SK9K4 (JX905358), *O.* sp. BS-3 (GU045476), bacterium OL-1 (LK021119), *O.* sp. J21 (DQ168838) and several clones]. Furthermore, it was found that the MGB probe had one mismatch with *O. profuse*, two mismatches with *O. uli* and *A. parvulum*, and three mismatches with *O. umbonata* (Fig. 1). The specificity of the primers was tested against *O. profusa*, *O. uli*, *O. umbonata*, *A. parvulum*, and a set of seven dominant pig gut microbial strains using either conventional PCR (Fig. 2a) or SYBR real-time PCR (Fig. 2b). The gDNA from *O. scatoligenes*, *O. profusa*, and *O. uli* was amplified in conventional PCR and SYBR Green real time PCR with expected size of 95 bp. gDNA from the other bacteria strains was not amplified. As shown in Fig. 2c, the use of the primer-MGB probe pair in TaqMan MGB real time PCR was specific for the detection of *O. scatologens* as it did not cross-react with gDNA from any of the other *Olsenella* species or the gastrointestinal isolates. The Ct difference between *O. scatoligenes* and non-target samples was equivalent to at least 2 logs of cell numbers.

### gDNA standard

**Fig. 3:**
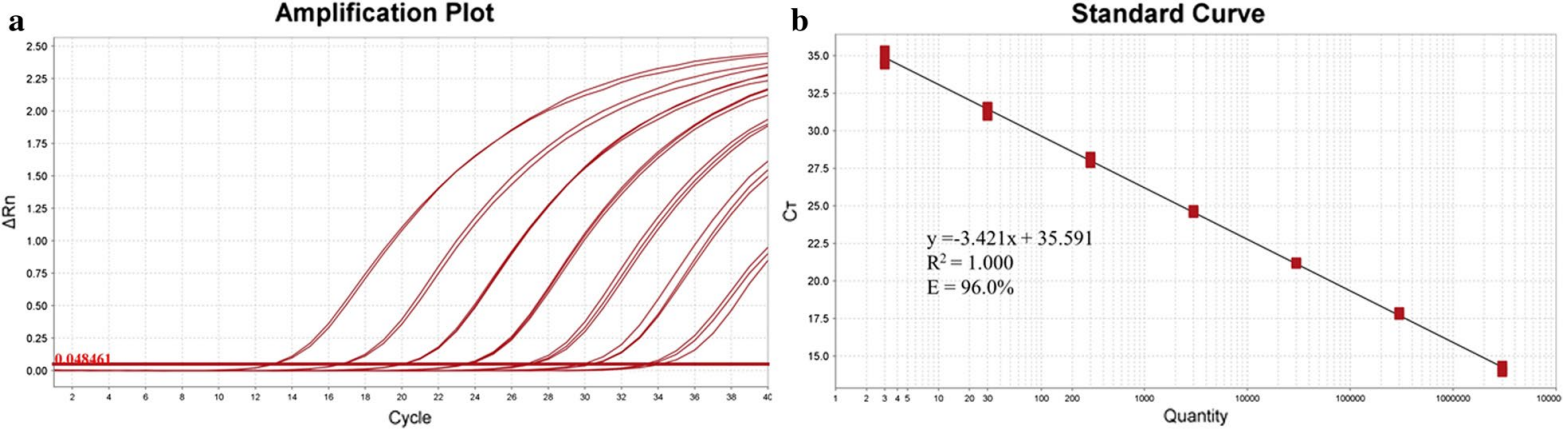
A representative amplification plot (**a**) and standard curve (**b**) of *O. scatoligenes* Taqman-MGB qPCR assay with tenfold dilutions of *O. scatoligenes* SK9K4^T^ genomic DNA ranging from 3 × 10^6^ to 3 × 10^0^ copies per reaction by using ViiA™ 7 RUO software version 1.2.1

Dilutions (ranging from 3 × 10^6^ to 3 × 10^0^ copies per reaction) of purified gDNA from SK9K4^T^ were used to construct standard curves and assess the probe sensitivity. The Ct values of the qPCR assay with MGB probe ranged between 13.26 (3 × 10^6^ copies) and 33.93 (3 × 10^0^ copies) (Table 2). The mean and standard deviation of efficiency of gDNA standard was 95.4 ± 0.5%, the R^2^ was 1.000 ± 0.000, and the slope was − 3.437 ± 0.014. A representative set of gDNA standard curve is shown in Fig. 3. The regression line for the curve was y = − 3.421 × + 35.591. The LOQ giving a reliable and reproducible signal was found to be 4.07 fg, which corresponds approximately to 3 genomic copies per reaction. The gDNA standard curve was used to obtain an absolute quantification of *O. scatoligenes* (Table 4).

**Table 2:** Reproducibility, intra-assay, and inter-assay coefficient of variation for TaqMan qPCR

No. of SK9K4^T^ genome (copies/reaction)	Intra-assay CT value	CV (%)	Inter-assay CT value	CV (%)
3 × 10^6^	13.30 ± 0.20	1.49	13.26 ± 0.10	0.74
3 × 10^5^	16.94 ± 0.07	0.42	16.86 ± 0.13	0.80
3 × 10^4^	20.33 ± 0.04	0.19	20.01 ± 0.48	2.40
3 × 10^3^	23.73 ± 0.09	0.37	23.62 ± 0.13	0.56
3 × 10^2^	27.17 ± 0.23	0.85	27.80 ± 0.12	0.58
3 × 10^1^	30.46 ± 0.32	1.05	30.38 ± 0.22	0.72
3 × 10^0^	33.93 ± 0.46	0.83	33.73 ± 0.25	0.73

### Reference standard

**Fig. 4:**
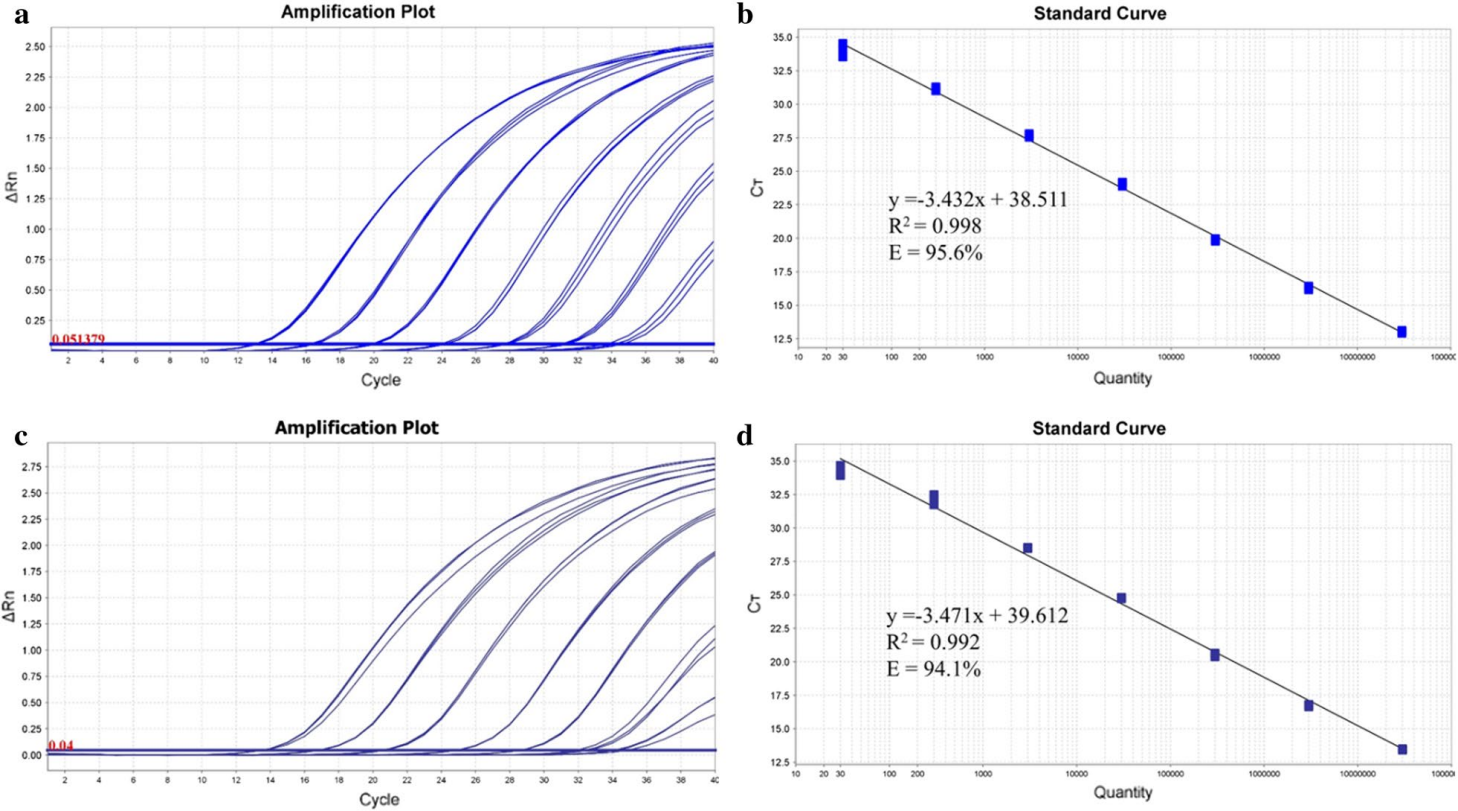
Amplification plots (**a**, **c**) and standard curves (**b**, **d**) of *O. scatoligenes* Taqman-MGB qPCR assay with tenfold dilutions of DNA from cecal (**a**, **b**) or colonic digesta (**c**, **d**) spiked with 1.5 × 10^10^ cells/g *O. scatoligenes* SK9K4^T^ (ranging from 3 × 10^7^ to 3 × 10^2^ cells per reaction) by using ViiA™ 7 RUO software version 1.2.1

The reference standards based on a dilution series of gDNA from cecal or colonic digesta spiked with 1.5 × 10^10^ cells/g *O. scatoligenes* SK9K4^T^ (ranging from 3 × 10^1^ to 3 × 10^7^ cells per reaction) showed a log-linear correction of 7 log units (Fig. 4). The regression lines for the reference standard curves of cecal and colonic samples were y = − 3.432 × + 38.511 (Fig. 4b) and y = − 3.471 + 39.612 (Fig. 4d), respectively. The R^2^ values of the regression lines were 0.998 (cecal samples) and 0.992 (colonic samples), and the amplification efficiencies determined from the linear regression curves were 95.6 and 94.1%, respectively. The LOQ for both cecal and colonical samples was 1.5 × 10^4^ cells/g (3 × 10^1^ cells per reaction) when using 10 ng of DNA extract in the real-rime PCR assay.

### Reproducibility

The reproducibility of the *O. scatoligenes* TaqMan qPCR assay was demonstrated by evaluating the variability of the Ct values obtained after amplification of tenfold serial dilutions of the gDNA standard ranging from 3 × 10^6^ to 3 × 10^0^ copies per PCR reaction in triplicate intra- and inter-runs (Table 2). The CV of the mean Ct values obtained for the gDNA standard curve ranged from 0.19 to 1.49% intra-assay and 0.56 to 2.40% inter-assay. The results demonstrated that the established TaqMan-MGB qPCR method was able to characterized *O. scatoligenes* within a wide range (7 log) with high precision.

### Spike recovery

The recovery of *O. scatoligenes* gDNA was measured on colonic digesta samples from three pigs, spiked with a known amount of reference strain cells (from 3.0 × 10^4^ to 3.0 × 10^7^ cells per PCR reaction). After DNA isolation, quantification and calculation, the recovery rate was found to be between 6.4 and 9.5% (Table 3). The gDNA recovery rate for digesta samples, spiked with lower amounts (≤ 3.0 × 10^3^ cells per PCR reaction) of *O. scatoligenes* was artificially elevated (data not shown) due to the background of native *O. scatoligenes* (around 10^5^–10^6^ cells/g digesta) in the colonic samples.

**Table 3:** DNA recovery rates from serially diluted *O. scatoligenes* SK9K4^T^ cells spiked into colonic digesta

Spiked^a^ (cells/reaction)	Detected^b^ (copies/reaction)	Recovery^c^ (%)
3.0 × 10^7^	(2.64 ± 0.45) × 10^6^	8.8 ± 1.5
3.0 × 10^6^	(2.86 ± 0.59) × 10^5^	9.5 ± 2.0
3.0 × 10^5^	(2.67 ± 0.42) × 10^4^	8.9 ± 1.4
3.0 × 10^4^	(1.92 ± 0.49) × 10^3^	6.4 ± 1.6

^a^Three 200 mg colonic digesta samples were spiked with 3 × 10^2^–3 × 10^9^ cells of SK9K4^T^, giving spiked concentrations at 3 × 10^0^ to 3 × 10^7^ cells per PCR reaction. Samples with values below 3.0 × 10^4^ cells per PCR reaction are not shown due to abundance of indigenous *O. scatoligenes* in colon

^b^Detected *O. scatoligenes* genomic copies/reaction was obtained from gDNA standard curve

^c^Recovery rate was calculated as the quotient of recovered copies and spiked cells

### Application of the TaqMan qPCR assay

The quantification of *O. scatoligenes* in pig gut digesta samples using qPCR was performed with the reference standards. Generally, higher numerical values of *O. scatoligenes* were measured in the chicory group than in the control group at both ages and in both sexes. However, the results were only significantly different for the young entire male pigs (Table 4). Further, *O. scatoligenes* number were higher in colonic digesta compared to cecal digesta (6.00 vs. 5.12 log cells/g). Moreover, higher numerical values were measured in males compared to females (5.25 vs. 4.92 log cells/g in cecal digesta; and 6.23 vs. 5.96 log cells/g in colonic digesta); and in young pigs compared to old pigs (5.52 vs. 4.71 log cells/g in cecal digesta; and 6.31 vs. 5.89 log cells/g in colonic digesta). Adding chicory roots to the diet significantly reduced (*p* < 0.001) both skatole and indole concentration in cecal and colonic digesta regardless of sex or age (Table 4).

**Table 4:** *Olsenella scatoligenes* (log cell/g digesta), skatole (mmol/kg digesta), and indole (mmol/kg digesta) concentrations in cecal and colonic digesta of pigs fed the control or Chicory

Diet^1^	Control				Chicory										
Sex	Female		Male		Female		Male		*p* value						
Weight (kg)	60 (n = 3)	120 (n = 2)	60 (n = 3)	120 (n = 4)	60 (n = 2)	120 (n = 3)	60 (n = 4)	120 (n = 3)	Diet	Sex	Weight	D*S	D*W	S*W	D*S*W
Caecum															
*O. scatoligenes*	4.84 ± 0.28^a^	4.74 ± 0.23^a^	5.02 ± 0.20^a^	4.73 ± 0.23^a^	5.38 ± 0.23^a^	4.61 ± 0.28^a^	6.77 ± 0.23^b^	4.74 ± 0.20^a^	0.005	0.023	< 0.001	0.061	0.003	0.047	0.135
Skatole	0.8 ± 0.9	2.4 ± 0.7	2.3 ± 0.6	2.9 ± 0.7	0.0 ± 0.7	0.0 ± 0.9	0.0 ± 0.7	0.0 ± 0.6	0.001	0.360	0.315	0.360	0.315	0.644	0.644
Indole	2.7 ± 1.6	2.9 ± 1.3	5.4 ± 1.6	3.7 ± 1.3	0.7 ± 1.3	1.6 ± 1.6	0.5 ± 1.3	0.4 ± 1.2	0.008	0.584	0.860	0.209	0.573	0.468	0.786
Colon															
*O. scatoligene*	6.01 ± 0.13^a^	5.76 ± 0.11^a^	5.93 ± 0.09^a^	5.83 ± 0.11^a^	6.12 ± 0.11^a^	5.97 ± 0.13^a^	7.23 ± 0.11^b^	6.02 ± 0.11^a^	< 0.001	0.002	< 0.001	0.002	0.006	0.010	0.002
Skatole	16.5 ± 5.3	29.4 ± 4.3	21.9 ± 3.7	24.7 ± 4.3	0.0 ± 4.3	1.0 ± 5.3	0.2 ± 4.3	0.2 ± 3.7	< 0.001	0.990	0.202	0.917	0.257	0.392	0.473
Indole	6.0 ± 1.9	7.7 ± 1.6	10.1 ± 1.4	5.1 ± 1.6	0.3 ± 1.6	1.2 ± 1.9	0.2 ± 1.6	0.3 ± 1.4	< 0.001	0.888	0.610	0.586	0.361	0.120	0.217

Values are least square means and standard error

^1^Experimental diets: control = a standard Danish grower diet based on wheat, barley, and soybean meal; chicory = the same diet in which barley and wheat had been substituted with 25% chicory root

^a, b^Means within a row without a common superscript letter differ (*p *< 0.05)

## Discussion

The use of TaqMan-MGB qPCR enabled specific enumeration of the skatole-producing bacterium *O. scatoligenes* in pig hindgut. One of the challenges in the development of qPCR assays is designing primers that specifically target the species of interest in samples containing closely related bacteria. So far, only one 16s RNA gene-based nested PCR protocol has been established to detect *Olsenella* species, yet this method has failed to discriminate *O. uli* from *O. profusa* (Rôças and Siqueira 2005). Our results (Fig. 2a, b) showed that conventional PCR and SYBR Green qPCR alone was not sufficient to distinguish *O. scatoligenes* from *O. profusa* and *O. uli*, due to high sequence homology in the variable regions of the 16s rRNA gene among the *Olsenella* species. Due to significantly improved hybridization properties of MGB probes (Kutyavin 2000), we thus opted to employ a TaqMan-MGB probe. The MGB stabilizes A/T rich duplexes, allowing to use shorter probes with higher melting temperature compared to ordinary DNA probes, and the increased specificity of an MGB probe allows discrimination with a single-base mismatch (Van Hoeyveld et al. 2004; Mingxiao et al. 2013). Therefore, in order to increase the specificity of the qPCR reaction for targeting *O. scatoligenes*, we designed a MGB probe with 1–3 mismatches to the closest related species. The Ct difference between *O. scatoligenes* and the closest related species, *O. profusa* was equivalent to at least two log units of cells. Thus, signals of unspecific targets in gut samples were considered not to compromise the specific enumeration of *O. scatoligenes*. Our results supported that MGB probes can be more sequence specific than unmodified 16S DNA probes, especially for single-base mismatches.

Efficiency and accuracy of qPCR depends on template DNA quantity and quality. Two main obstacles for obtaining sufficient and good quality 16S DNA are inefficient recovery of total gDNA from the targets in question and presence of PCR inhibitory compounds from the environmental matrix (Zoetendal et al. 2001). Incomplete lysis of the bacteria in a sample reduces the number of genomic copies available for PCR and the presence of inhibitors reduces the efficiency of the amplification (Coyne et al. 2005). Potential inhibition of qPCR can be evaluated by performing a serial dilution of the DNA extract prior to performing PCR. Presence of inhibitory substrates in digesta samples is possible but in the present study it was considered to be a minor problem, as shown by the relatively high amplification efficiencies (around 95%) of the reference standards.

The low recovery of DNA from digesta samples was expected and seems difficult to avoid, as demonstrated by a study using different DNA extraction methods for analysis of cecal microbiota, resulting in 64–99.9% loss of the retrievable DNA (Scupham et al. 2007). The bacterial population of the pig cecum and colon is numerically large, with at least 10^10^ cells/g wet digesta [bacteria numbers in the colon are typically twice the number in the cecum (Butine and Leedle 1989)], resulting in a DNA mass ≥ 32 µg/g wet digesta (calculated from the density of the bacteria, ~ 3 Mbp as an average genome size and 1.09 × 10^−21^ g as the mass of DNA per genome). However, in our study, using the Qubit^®^ fluorometer method, only 1.0 and 5.1 µg total gDNA/g were recovered from cecal and colonic digesta, respectively. The low recovery of gDNA extraction was also confirmed by the DNA recovery assay. Less than 10% of *O. scatoligenes*-specific DNA was detected after DNA isolation and subsequent amplification in our study. In line with our result, Nathues et al. (2009) reported, using QIAamp^®^ DNA Stool mini Kit, that only 3.5% of the specific DNA added was recovered from spiked feces. The commercial QIAamp^®^ DNA Stool kit has shown high extraction efficiency and PCR-compatibility, and is frequently used in the extraction of gDNA from human feces (McOrist et al. 2002; Nechvatal et al. 2008; Salonen et al. 2010). However, another study showed that this kit was less efficient for the extraction of DNA from pig manure samples compared to other commercial kits (Desneux and Pourcher 2014). The QIAamp^®^ kit uses a chemical lysis procedure that may lead to incomplete cell lysis. Furthermore, after comparing several DNA extraction methods, FastDNA^(^™^)^ SPIN Kit for Soil (MP Biomedical) was recommended by Burbach et al. (2016) as a suitable DNA extraction kit for the analyses of porcine gastrointestinal tract samples. Moroever, the low gDNA recovery in our study may partly be caused by long-term (~ 2 years) storage of digesta samples at − 20 °C. Degradation of DNA by endonucleases most likely happens during the long-term storage at − 20 °C, resulting in reduced DNA yield (Metzler-Zebeli et al. 2016). Optimizing sample storage (e.g. lyophilization) and DNA extraction methods (e.g. including a step for the mechanical disruption of microbial cells by bead beating) are therefore recommended (Yuan et al. 2012).

In qPCR assays, a plasmid or gDNA carrying the target genes is most commonly used as the standard. This method is relatively straightforward. However, the amplification efficiencies and DNA recovery efficiencies of plasmids/gDNA or pure cell cultures, used for generation of a standard curve, may not be similar to that of the DNA extracted from environmental samples. In order to overcome this problem, a known concentration of target microbe cells was spiked into the digesta samples as a reference standard. Since DNA is extracted from a matrix in the presence of a known amount of spiked target microbe cells, used as a reference standard, inherent variability in extraction and amplification efficiencies is taken into account by this procedure (Coyne et al. 2005). To obtain a more precise determination of microbes of interest in a complex sample, there are increasing numbers of studies using cell-spiked samples as reference standards (Abildgaard et al. 2010; Hariganeya et al. 2013; Sattler et al. 2014). According to our results, applying a pure-culture gDNA standard to estimate *O. scatoligenes* in gut samples would lead to a more than tenfold under-estimation of cell numbers in the gut digesta due to the low DNA recovery efficiency. Therefore, cell-spiked digesta have to be used as reference standards for enumeration of *O. scatoligenes* in gut digesta samples.

In some qPCR studies, standard curves have been made by inoculating a sample matrix, free of the target microbe, with serial dilutions of target microbe cells, obtaining detection limits of around 10^2^–10^3^ (Abildgaard et al. 2010) or 10^3^–10^4^ cells/g matrix (Wattanaphansak et al. 2010). The drawback of this approach is that a series of reference matrix samples are spiked independently with a dilution series of target microbe cells, potentially introducing another source of error owing to inconsistent DNA extraction efficiency of these samples (Coyne et al. 2005). In our study, the gut digesta samples were spiked with a single, defined concentration of target microbe cells after which DNA was isolated and then serially diluted. Sattler et al. (2014) claimed that use of the latter approach may lead to a relatively low detection limit of 10^1^–10^2^ cells/g matrix. Since *O. scatoligenes* is a common member of the pig gut microbiota, it may however not be possible to obtain of *O. scatoligenes*-free digesta samples, compromising the accuracy of the standard curve for low cell numbers.

The pure culture gDNA standard, which was used to obtain an absolute quantification of *O. scatoligenes* gDNA, had a quantification limit of 3 copies per PCR reaction. However, the detection limit was only 10^3^ cells/g when using spiked digesta as reference standards owing to poor DNA recovery efficiency as well as the above mentioned issue of the native background *O. scatoligenes* population.

The developed qPCR assay could detect the presence of *O. scatoligenes* at 10^2^ cells/g of digesta, but was unable to quantify the population precisely for *O. scatologenes* levels as low as this. However, the developed qPCR assay had a high level of reproducibility, as shown by relatively low intra-assay and inter-assay coefficients of variation.

Little is known about the presence and identity of skatole-producing bacteria in the hindgut of pigs and how these bacteria are affected by fermentable dietary fibre such as inulin from chicory roots. According to Kim et al. (2011), analyzing the microbiota composition by pyrosequencing, the *Olsenella* genus constitutes 0.12% (from 0.02 to 0.94%) of the total microbiota in pig feces. Using the most probable number technique in combination with skatole measurements, it has been estimated that the population of skatole-producing bacteria accounts for < 0.01% of the total microbiota in pig fecal samples (Jensen and Jensen 1998). In a previous study, we screened 122 isolates, representing unique 122 OTUs in a collection of 2678 isolates from the pig GI-tract, for their ability to produce skatole from TRP or IAA. The results showed that only one of these isolates, *O. scatoligenes* SK9K4^T^, was able to produce skatole (Li et al. 2015). *Megasphaera* sp. strain TrE9262 has been reported to produce skatole (Attwood et al. 2006). However, the isolate *Megasphaera* strain DJF_B143 from our collection, which has 99.9% 16S rRNA gene similarity with strain TrE9262, did not produce skatole (Li et al. 2016b). Therefore, we suspected that skatole-producing bacteria were limited only to a few species, with *O. scatoligenes* being the main dominant skatole-producing bacterium in the pig GI-tract. In the current study, we found that *O. scatoligenes* accounted for < 0.01% (10^5^–10^6^ cells/g) of the total population of the hindgut microbiota, which is in agreement with the findings of Jensen and Jensen (1998). An increased proliferation of the *Olsenella* genus by dietary fiber has been observed in some studies (Haenen et al. 2013; Mao et al. 2015). Interestingly, in the current study, dietary addition of chicory roots only significantly increased the abundance of *O. scatoligenes* in ~ 60 kg entire male pigs.

A reduction of skatole production in the pig hindgut by adding the inulin rich feed component chicory root to the diet has been reported (Wesoly and Weiler 2012; Zammerini et al. 2012), and was also observed in the present study. Three main underlying mechanisms behind this effect have been proposed in the literature: (i) a high content of fermentable fiber in the hindgut increases the microbial activity, resulting in more tryptophan incorporated as bacterial biomass and thereby leaving less substrate for skatole production (Jensen et al. 1995; Xu et al. 2002); (ii) an increased butyrate production, due to inulin-type fructans in the diet (Falony et al. 2006), reduces sloughing of enterocytes, whereby less endogenous tryptophan from cell debris is available for skatole production (Claus et al. 2003). Both of these mechanisms result in a reduced level of tryptophan as substrate for skatole production. Therefore, (iii) an altered gut microbiota characterized by a lower abundance of skatole-producing bacteria could be expected when adding high levels of dietary fermentable fiber. However, the present study demonstrated that the skatole-reducing effect of chicory root amended-diets is apparently not by inhibiting the growth of skatole-producing bacteria in the pig hindgut. Contrarily, the use of chicory root amended-diets seemed to increase *O. scatoligenes* proliferation, though this was only significant in young entire male pigs.

In conclusion, the present study demonstrated, for the first time, reliable and specific quantification of *O. scatoligenes* in pig gut content samples by use of a developed TaqMan^®^-MGB real-time PCR assay. The assay was shown to have high specificity, sensitivity, accuracy and reproducibility. With the assay, we showed that a reducing effect of chicory root amended-diets on skatole production in the pig hindgut was not associated with a reduced number of *O. scatoligenes*. Thus, the abundance of *O. scatoligenes* in the hindgut of pigs does not, in itself, seem to be a valid indicator of boar taint.

## Additional file

**Additional file 1: Table S1.** Ingredients and analyzed composition of the experimental diets.

## Abbreviations

TRP: l-tryptophan
IAA: indol-3-acetic acid
GI-tract: gastrointestinal tract
MGB: minor groove binder
DSMZ: German collection of microorganisms and cell cultures
gDNA: genomic DNA
BLASTn: basic local alignment search tool
RDP: ribosomal database project
LOQ: limit of quantification
CV: coefficient of variation
