# Supplementary material for: Development of a species-specific TaqMan-MGB real-time PCR assay to quantify Olsenella scatoligenes in pigs offered a chicory root-based diet
Source: AMB Express. 2018 Jun 16;8:99. doi: 10.1186/s13568-018-0627-y (PMC6004268; doi:10.1186/s13568-018-0627-y)
Supplement: Supplementary file 1 — Additional file 1: Table S1. Ingredients and analyzed composition of the experimental diets. [file 13568_2018_627_MOESM1_ESM.pdf]

AMB Express

Development of a species-specific TaqMan-MGB real-time PCR assay to quantify *Olsenella scatoligenes* in pigs offered a chicory root-based diet

Xiaoqiong Li, Bent Borg Jensen, Ole Højberg, Samantha Joan Noel, Nuria Canibe*

Department of Animal Science, Faculty of Science and Technology, Aarhus University, Blichers Allé 20, P. O. Box 50, DK-8830 Tjele, Denmark

*Corresponding author: Nuria Canibe   Nuria.Canibe@anis.au.dk   +45 87 15 80 58

Table S1 Ingredients and analyzed composition of the experimental diets.

**Ingredient composition (%)**	Control/Butyrate	Chicory
Wheat	55.97	28.84
Barley	20.00	20.00
Dehulled toasted soybean meal	9.93	12.00
Dehulled sunflower cake	8.00	8.00
Dried milled chicory root[a]	0.00	25.00
Soybean oil	1.70	1.40
Sugar beet molasses	1.50	2.00
Calcium carbonate	1.42	1.33
Monocalcium phosphate	0.36	0.35
Sodium chloride	0.49	0.48
L-Lysine-HCl, 98%	0.32	0.29
DL-Methionine , 98%	0.03	0.04
Threonine, 98%	0.08	0.08
Vitamin and mineral premix[b]	0.20	0.20
**Analyzed composition (g/kg dry matter)**		
DM	866	778
Fat	34	28
Crude protein	154	157
Ash	42	54
Calcium	6.01	6.65
Phosphorus	4.31	4.13
Valine	7.00	6.79
Cystein + Cystine	3.29	2.88
Lysine	8.88	9.33
Methionine	2.76	2.80
Threonin	5.62	6.16

[a] The content of fructan in the dried milled chicory root was 65.2 %.

[b] Providing the following per kilogram of diet: 3696 IU of vitamin A; 370 IU of vitamin D_3; 61 mg of vitamin E; 1.8 mg of vitamin K_3; 1.8 mg of vitamin B_1; 1.8 mg of vitamin B_2; 2.8 mg of vitamin B_6; 0.02 mg of vitamin B_{12}; 9.2 mg of Ca-D-pantothenic acid; 18.5 mg of niacin; 0.05 mg of biotin; 55 mg of DL-alpha-tocopherol; 61 mg of DL-alpha-tocopherol acetate; 74 mg of Fe (Fe (II) sulphate); 13 mg of Cu (Cu(II) sulphate) and 37 mg Mn (Mn(II) oxide).
